# The cellular basis of organ failure in sepsis—signaling during damage and repair processes

**DOI:** 10.1007/s00063-020-00673-4

**Published:** 2020-03-31

**Authors:** M. Bauer, R. Wetzker

**Affiliations:** 1 Department of Anesthesiology and Intensive Care Medicine, Jena University Hospital, Am Klinikum 1, 07747 Jena, Germany; 2 Integrated Research and Treatment Center Center for Sepsis Control and Care (CSCC), Jena University Hospital, Jena, Germany

**Keywords:** Sepsis, Resistance, Immunopathology, Disease tolerance, Immunoparalysis, Sepsis, Resistenz, Immunpathologie, Toleranz, Immunparalyse

## Abstract

Sepsis is defined as life-threatening organ dysfunction caused by a dysregulated host response to infection. This definition, updated in 2016, shifted the conceptual focus from exclusive attention to the systemic inflammatory response toward the multifactorial tissue damage that occurs during the progression of infection to sepsis and shock. Whereas targeting the inflammatory host response to infection did not translate into improved clinical management of sepsis, recent findings might shed new light on the maladaptive host–pathogen interaction in sepsis and pave the way for “theranostic” interventions. In addition to the well-known resistance responses of the immune system that result in pathogen clearance, “disease tolerance” has recently been acknowledged as a coping mechanism of presumably equal importance. We propose that both defense mechanisms, “resistance” and “disease tolerance”, can get out of control in sepsis. Whereas excessive activation of resistance pathways propagates tissue damage via immunopathology, an inappropriate “tolerance” might entail immunoparalysis accompanied by fulminant, recurrent or persisting infection. The review introduces key signaling processes involved in infection-induced “resistance” and “tolerance”. We propose that elaboration of these signaling pathways allows novel insights into sepsis-associated tissue damage and repair processes. Moreover theranostic opportunities for the specific treatment of sepsis-related hyperinflammation or immunoparalysis will be introduced. Agents specifically affecting either hyperinflammation or immunoparalysis in the course of sepsis might add to the therapeutic toolbox of personalized care in the field of organ dysfunction caused by infection. (This article is freely available.)

## Abstract (de)

Sepsis ist definiert als lebensbedrohliche Organfunktionsstörung infolge einer gestörten Wirtsantwort auf eine Infektion. Diese Definition, 2016 aktualisiert, verlagerte den konzeptionellen Fokus von der Entzündungsreaktion des Wirts zur multifaktoriellen Gewebeschädigung, die während des Fortschreitens der Infektion zur Sepsis auftritt. Während die therapeutische Intervention in die Entzündungsreaktion des Wirts auf eine Infektion nicht zu einem verbesserten klinischen Management der Sepsis führte, werfen jüngste Erkenntnisse ein neues Licht auf die maladaptive Wechselwirkung zwischen Wirt und Krankheitserreger bei der Sepsis. Zusätzlich zu den bekannten Resistenzreaktionen des Immunsystems, die zur Beseitigung von Krankheitserregern führen, wird zunehmend die „disease tolerance“ oder „Resilienz“ als ein Bewältigungsmechanismus von vermutlich ähnlicher Bedeutung erkannt. Wir diskutieren im vorliegenden Review, dass sowohl die „Resistenz“ als auch die „Resilienz“ bei Sepsis außer Kontrolle geraten können. Während eine übermäßige Aktivierung von Resistenzreaktionen zur Immunpathologie mit konsekutiver Gewebeschädigung führt, kann eine unangemessene Resilienz wahrscheinlich in eine Immunparalyse mit fulminanten, rekurrierenden oder persistierenden Infektionen münden. Wir diskutieren zudem wichtige Signalprozesse, die an der Vermittlung infektionsbedingter Resistenz- und Resilienzmechanismen beteiligt sind. Wir denken, dass diese Signalwege neue Möglichkeiten zur Therapie der sepsisassoziierten Gewebeschädigung und zur Stimulation von Reparaturprozessen eröffnen. Darüber hinaus werden neue Ansätze zur gezielten Behandlung von sepsisbedingter Hyperinflammation oder Immunparalyse vorgestellt, die das Portfolio für die personalisierte Versorgung der durch Sepsis verursachten Organfunktionsstörung erweitern sollten.

## Introduction

Infections reflect frequent and typically self-limiting events. A proportion of infected patients, however, develop organ dysfunction through a dysregulated host response to infection, i.e., sepsis [40]. Research aimed to elaborate the defense mechanisms against infections, clearly focused on lowering the pathogen burden through *resistance*, i.e., the ability of the organism to detect and to destroy the invading microorganisms. During the past years an alternative defense mechanisms came into focus: *Disease tolerance*, mainly characterized by regenerative responses, which allow retaining cell and organ functions of the infected host without lowering the pathogen burden.

## Failing resistance and tolerance in sepsis

An overwhelming resistance response of the immune system in sepsis has been hypothesized for a century and found a convincing molecular explanation in 1985 by Bruce Beutler and colleagues [7]: They observed tissue damage in sepsis and firstly interpreted this phenomenon as an overreaction of immune cells using their antibacterial effector molecules leading to “collateral damage”. Understanding of sepsis as a systemic (hyper-)inflammatory response (SIRS) to pathogenic infections prevailed the interpretation of sepsis-associated organ dysfunction for the last 3 decades.

During the past years reinterpretation of sepsis pathogenesis was provoked by a phenomenon occurring in later stages of the disease. The majority of patients who survive the initial phase of sepsis suffer from impaired innate and acquired immunity up to the occurrence of secondary infections or reactivation of dormant viruses [8].

There is increasing evidence that this phenomenon might be interpreted as uncontrolled tolerance. The normally balanced relation of damaging effects of microbial pathogens in host tissue and the associated maintenance responses seemingly go out of control. The repair capacity of the affected organs becomes increasingly exhausted.

**Fig. 1:**
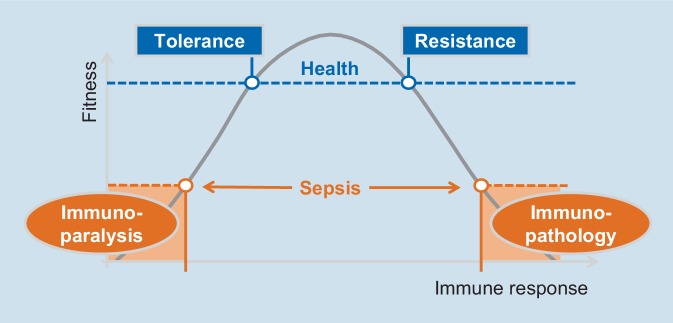
Fitness of the host and immune response to infections

Hence both failing resistance and tolerance responses contribute to the phenotypes of sepsis (Fig. 1). Notably, in septic patients evidence for all aspects of immune dysfunction, i.e., predominant hyperinflammatory responses (“SIRS”), patients with predominant immunoparalytic characteristics (“CARS”) and also mixed patterns (“MARS”) have been reported [8, 19, 33].

The seminal role of resistance and tolerance in the adaptive responses of higher organisms to infectious attacks raises the question for the origin and causes of these differential response patterns, in particular when the host is repeatedly confronted with pathogens and their pathogen-associated molecular patterns (PAMPs).

Many data indicate differential ability of specific pathogens or PAMPs to induce alternative immune responses. Thus ample studies reveal lipopolysaccharides (LPS) as constituents of gram negative bacteria as typical inducers of decreased release of proinflammatory cytokines from affected immune cells unveiling LPS as typical mediators of (endotoxin) *tolerance *[45]. Vice versa β‑glucan as a component of fungal cell membranes has been frequently shown to induce enduring increase of the release of proinflammatory cytokines from immune cells, characterizing β‑glucan as prototypical inducer of *resistance* responses [32].

This simple scheme is considerably challenged by recent observations of consecutive appearance of resistance and tolerance responses in dependence on the concentration of PAMPs. For instance, Maitra et al. revealed increased resistance responses of monocytes after treating monocytes with very low doses of LPS [27]. Similar stimulation of proinflammatory cytokine release at extremely low doses of LPS—i.e. increased resistance—and inhibition at high LPS doses—i.e. tolerance—has been shown in a study of microglia response pattern to PAMPs [21]. Consequently, both pathogen species and pathogen dose might determine resistance or tolerance responses of the host [6].

The complex differential response patterns of the immune system and other organs during sepsis necessitate careful clinical monitoring, i.e. personalization of care. While already a reality in the field of oncology, the time critical decisions in intensive care medicine have so far hindered to take functional characteristics of resistance and tolerance responses of the immune system into account. Thus, the need for better diagnostic tests has been voiced for infectious diseases in general and with special emphasis on sepsis [15] and seems feasible with modern diagnostic platforms. We and others have shown that compound biomarker panels that consist of several transcripts reflecting both the proinflammatory and anti-inflammatory response can be used at the point-of-care to provide additional information on the host response compared to conventional biomarkers [4, 30] allowing to discriminate various phenotypes of sepsis. In addition, recent evidence would suggest that pattern recognition with artificial intelligence on routine parameters might help to discriminate inflammatory or metabolic phenotypes with potential impact on treatment [38].

## Metabolic reflections of resistance and tolerance responses

The biological phenomena of resistance and tolerance responses of the immune system are closely linked to energy resources and metabolic processes in the cells involved. In general resistance responses reveal anabolic, i.e. energy-consuming metabolic traits, whereas tolerance predominantly implicates catabolic, i.e. energy providing processes [6].

The energy-consuming character of anabolic resistance responses can be illustrated by the resource needs of the clonal expansion of adaptive immune cells secondary to infection. Doing so, the immune system and other organs involved in the resistance response strongly compete for energy resources with other anabolic functions.

In contrast to the energy wasting resistance responses, immune tolerance and in a broader sense organismic tolerance represents an energy saving response pattern of the organism to infection [2]. During tolerance responses the titer of invading microorganism does not change significantly and cells and organs involved tend to react passively by building barriers for microorganism dissemination and/or regenerating cells and their organelles, which are damaged by the pathogens [2].

Recent studies provide evidence that overwhelming immune tolerance leads to immunoparalysis (Fig. 1). Experimentally, Grondman et al. showed tight association of endotoxin-induced immune tolerance with loss of monocyte metabolic plasticity and reduction of oxidative burst with leading to microbial dissemination [18].

It becomes apparent in all these studies that metabolic processes are closely interlinked to damage and repair processes in cells and tissues [5]. The homeostasis of synthesis and formation of cellular organelles on one site and their degradation is an essential prerequisite for vitality of the cells and, thus, integrity of tissue and organs.

## Signaling and targeting of resistance and tolerance

Efficient adaptation to environmental challenges is key for survival and conserved throughout evolution. Consequently anabolic resistance- and catabolic tolerance responses need tight linkage to the energy metabolism of cells involved. To manage this goal all cells possess sensors for the energy state, most notably mechanistic target of rapamycin (mTOR) and AMP-activated protein kinase (AMPK) [6]. Both ubiquitous signaling proteins have been evolved during evolution over the last 2 billion years as key mediators of metabolic responses [37].

Notably, mTOR acts as a go-between the growth promoting effects of insulin. In doing so mTOR works in tight cooperation with its upstream mediators PI3K and Akt [35]. Rapamycin, a drug which directly interacts with mTOR, blocks its signaling effects and was shown to exhibit antiproliferative effects specifically on immune cells leading to its approval as immunosuppressive drug [42]. Consistent with the aforementioned concept, recent clinical studies reveal a significant decrease in inflammation and senescence markers in patients after treatment with mTOR inhibitors [24].

Some of the regenerative effects of rapamycin might be explained by its strong ability to induce autophagy [26]. Autophagy has been defined as stress response pathway mediating the breakdown of malfunctioning cellular material and initiating cell content recycling [36]. The central mediator of cellular recycling activities is AMPK [43].

AMPK acts as highly conserved signaling sensor activated by elevated AMP/ADP concentration [31]. Consequently, AMPK is activated by caloric restriction and deactivated by nutritional overload opposing mTOR, which is stimulated at nutrient abundance and anabolic conditions [37]. AMPK can also be activated by a number of energy-consuming stress responses including physical activities and infection [13]. Thus low doses of ionizing radiation were shown to induce a radioprotective effect by promoting DNA repair and cell survival signaling proteins through activating AMPK and inhibiting mTOR signaling [46].

Several studies suggest AMPK as a possible drug target in aging. Thus, treatment with metformin, an AMPK activator, can extend lifespan in several species, e.g. *Caenorhabditis elegans* and mice [14, 28]. Because of its life-extending effects in several species, as well as its anti-inflammatory and antidiabetic activities, metformin, along with rapamycin, has been approved for the clinical trial targeting aging with metformin (TAME) in elderly (clinicaltrials.gov).

mTOR has been characterized as a potent inhibitor of autophagy [1]. Vice versa AMPK can inhibit the activity of mTOR complex (mTORC1) via two different mechanisms, either by directly phosphorylating Raptor, a regulatory component of mTORC1, or by the phosphorylation of tuberous sclerosis protein 2 (TSC2) [25]. High fat diet and obesity lead to increased inflammation, macrophage activation and downregulation of AMPK [17]. Increased inflammation is one of the main causes of tissue deterioration with age. As previously discussed, activation of AMPK can reduce oxidative stress, decrease endoplasmatic reticulum stress and inflammation [20]. Notably, AMPK activation suppresses the signaling of anabolic insulin/IGF‑1 pathway underlining again the opposing functions of mTOR and AMPK in the control of anabolic and catabolic processes [35].

**Fig. 2:**
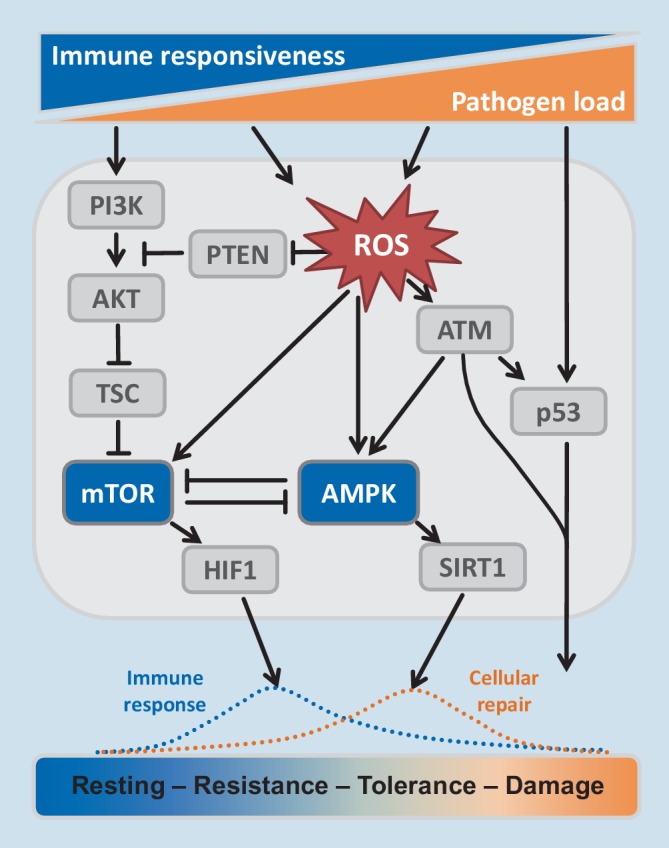
Signaling scheme reflecting signaling cascades of resistance and tolerance as a function of pathogen burden. Adapted from [6]

The opposing functions of mTOR and AMPK in the control of resistance and tolerance responses can be summarized in a signaling scheme, which also involves mediators “upstream” and “downstream” of these central sensors of cellular energy level (Fig. 2).

The predominant role of (pathogen) stress dose on the development of resistance and tolerance responses of immune cells has been outlined. Increasing pathogen stress is progressively exhausting energy resources of the immune system leading to decreasing “immune responsiveness” and concomitant tolerance responses. All stressors are inducing adaptive responses, which provoke energy deficit.

## How can these concepts be applied in sepsis and organ failure?

Recent observations in rodent sepsis models revealed novel therapeutic approaches to modulate the opposing phenotypes of altered immunity in sepsis. Probably the most surprising impulses to treat sepsis came from experiments investigating cross effects of environmental stressors on the response patterns of septic mice.

In a seminal study Figureido et al. [16] screened a large number of chemicals, including anthracyclines, for their effects on inflammatory reactions of a macrophage cell line. These cytotoxins—commonly applied for the therapy of solid cancers—were injected into mouse models of polymicrobial sepsis. In comparison to the controls, a significantly lower mortality of the toxin-treated animals was observed. Interestingly, whole body irradiation produced similar effects. More detailed investigations revealed stimulation of DNA repair mechanisms in response to trace amounts of anthracyclines with induction of autophagy. Together these data indicate that stressors like toxins or irradiation induce repair mechanisms, which suppress immunopathology thus preventing tissue damage. Interestingly, dietary restriction as another environmental stressor could suppress hyperinflammatory responses in septic mice [41]. Sepsis-induced mortality could be abolished by three weeks of moderate fasting prior to provoking sepsis by a polymicrobial cocktail or by LPS injection. In addition to conventional use of anti-inflammatory drugs different kinds of stressors might be applied to suppress inflammation-induced damage.

The anti-inflammatory effects of anthracylines, of radiation and food deprivation can be explained as a consequence of their energy-demanding effects. Environmental stressors and inflammatory reactions inside the immune cells compete for the same energy sources and consequently protein synthesis and cell proliferation. It can be supposed that treatment of cells with anthracylines and radiation or food deprivation provoke a turn of the immune responses towards tolerance reactions. Morbidity induced by systemic inflammation decreases and the organism exhibits “disease tolerance”.

What concepts arise regarding immunoparalysis and damage induced by disseminating microbes? There are initial hints that proinflammatory cytokines can be used to induce arousal of a “dormant” immune system. Leentjens et al. demonstrated partial reversion of immunoparalysis of humans by treatment with IFNγ in vivo [22]. The immunostimulatory agents used in these models cover a broad spectrum of agents from *Candida albicans* and its cell wall component β‑glucan [32], BCG vaccine [11] and surprisingly also acetylsalicylic acid (ASA) [23].

**Fig. 3:**
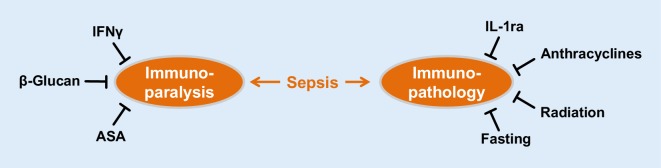
Evolving therapeutic options to modulate resistance and tolerance responses in sepsis models. *ASA* acetylsalicylic acid, *IFNγ* Interferon γ, *IL-1ra* Interleukin-1 receptor antagonist

An overview regarding opportunities to modulate immunity as it relates to cellular energy stores is shown in Fig. 3. In addition to direct immune-modulating agents, environmental stressors like low doses of cytotoxic agents, radiation or deprivation of food seem to enter translational research and could potentially explain observations in clinical trials, e.g. regarding the impact of caloric restriction on outcome in the ICU [10].

Taken together, interventions into anabolic and catabolic reactions in the course of sepsis might add to the therapeutic toolbox of personalized care in the field of infection-driven organ dysfunction.

## Outlook: Resistance and tolerance responses beyond the immune system

Evidently, resistance and tolerance responses are not restricted to the immune system. All other organs specifically respond to microbial infections. Tissue macrophages roving around the parenchyma act as sensors for invading microorganisms. Following interaction with the microbes they release signals which provoke defense reactions of the surrounding tissue [29]. Also non-immune cells have been shown to directly express *resistance* responses by attacking and destroying invading microbes. Most prominently antimicrobial peptides released by epithelial and other parenchymal cells exhibit the ability to destroy bacteria [44]. Ongoing efforts in experimental medicine are directed to use the antimicrobial capacity of peptides like defensins to treat specific infectious diseases [3]. The defense armory of parenchymal cells includes in addition phagocytosis of microbes and degradation by specific release of proteolytic enzymes or reactive oxygen species [12]. *Tolerance* to microbial infections is a widespread trait of parenchymal cells and organs. Originally explored in plants, tolerance responses have also been in the meantime detected in most of the parenchymal organs of mammals [2]. As a prototypical example accumulation of labile heme in plasma and urine during *Plasmodium* infection has been shown to induce pronounced disease tolerance to malaria. Specifically, the heme-induced tolerance response prevents the development of acute kidney injury, a clinical hallmark of severe malaria [34].

How do parenchymal organs cope with resistance and tolerance responses of the immune system? Relevant investigations reveal a tight overlap of pathogen induced metabolic and signaling reactions in immune and in parenchymal cells [12]. Unquestionably energy resources of all organs including the immune system are closely interlinked by the hormonal control of metabolites via the blood stream. Full supply of the organism with nutrients enables fervent resistance responses of the immune system but also high anabolic and proliferative capacity of parenchymal tissue. In contrast, calorie and energy deprivation might cause tolerant immune responses and catabolic maintenance reactions of the parenchyma.

In general, full regeneration of damaged tissue needs a well-balanced interplay of catabolic and anabolic events. In a first step damaged and functionless biomolecules and cellular organelles have to be degraded. Controlled proteolysis by intracellular proteasomes and extracellular proteases may take care of dysfunctional proteins [9]. Whole organelles are taken up and degraded by autophagy [36]. To fully regenerate damaged tissue these prototypical catabolic processes are mostly followed by backfilling of injured cells [39]. Here energy demanding proliferation of the surrounding tissue is necessary.

Thus, the precise adjustment of catabolic and anabolic processes is prerequisite for full regeneration of parenchymal tissue and all organs damaged either by hyperinflammation or microbial virulence in sepsis. In fact, host–pathogen interaction in sepsis comprises responses of parenchymal organs as a central, yet underestimated component. The highly dynamic tripartite interaction of pathogen, immune system and parenchyma seemingly defines the course of infection-related organ failure, i.e., sepsis and opens novel treatment options.

## Practical conclusion

- Organ dysfunction results from impaired adaptation to infection which is not restricted to “hyperinflammation”.
- The ability to adapt to the “pathogenic load” associated with infection is crucial for an organism and might involve “resistance” (i.e. responses of the immune system that lower pathogen burden) and “resilience” or “disease tolerance” (i.e. responses that increase the ability to cope with a persistent pathogen burden).
- Newly emerging treatment concepts that take into consideration opposing responses of immunity (“theranostics”) are promising but require diagnostic tests that allow identifying various phenotypes of sepsis at the point of care.
- These strategies are currently subject to clinical trials and require more evidence for translation into clinical practice.
